# Digital Technologies in the Surgical Treatment of Post-Traumatic Zygomatico-Orbital Deformities

**DOI:** 10.17691/stm2020.12.3.07

**Published:** 2020-06-28

**Authors:** N.E. Khomutinnikova, E.A. Durnovo, Yu.V. Vyseltseva, R.O. Gorbatov

**Affiliations:** Associate Professor, Department of Surgical Dentistry and Maxillofacial Surgery with the Course of Plastic Surgery; Privolzhsky Research Medical University, 10/1 Minin and Pozharsky Square, Nizhny Novgorod, 603005, Russia; Professor, Head of the Department of Surgical Dentistry and Maxillofacial Surgery with the Course of Plastic Surgery; Director of the Institute of Dentistry; Privolzhsky Research Medical University, 10/1 Minin and Pozharsky Square, Nizhny Novgorod, 603005, Russia; Associate Professor, Department of Surgical Dentistry and Maxillofacial Surgery with the Course of Plastic Surgery; Privolzhsky Research Medical University, 10/1 Minin and Pozharsky Square, Nizhny Novgorod, 603005, Russia; Associate Professor, Department of Traumatology, Orthopedics and Neurosurgery; Head of the Laboratory of Additive Technologies; Privolzhsky Research Medical University, 10/1 Minin and Pozharsky Square, Nizhny Novgorod, 603005, Russia

**Keywords:** orbital deformity, zygomatico-orbital complex, post-traumatic deformity, enophthalmos, orbital reconstruction, facial implants, digital technologies in the treatment of facial injuries, three-dimensional modeling of implants.

## Abstract

**The aim of the study** was to determine the efficacy of using digital technologies in patients with post-traumatic deformities of the zygomatico-orbital complex (ZOC) by comparing the results with the conventional methods of surgical treatment.

**Materials and Methods.:**

The article summarizes treatment results of 231 patients with ZOC injuries who underwent surgery at the clinical facilities of Privolzhsky Research Medical University (Nizhny Novgorod) in 2011–2019. There were treated 44.2% (102/231) of patients with post-traumatic deformities of ZOC, including 38.2% (39/102) with post-traumatic defects and deformities of the orbital floor.

Based on clinical and radiological planning of surgical operations, the patients were divided into two groups: group 1 included patients who underwent surgery without preoperative virtual planning (54.9% (56/102) of cases), group 2 included patients who underwent virtually planned surgical interventions (45.1% (46/102) of cases). There were 22 and 17 patients with orbital deformities in groups 1 and 2, respectively.

**Results.:**

The optimal restoration of ZOC anatomy was observed in 75% (42/56) of patients in group 1 and 93.5% (43/46) of patients in group 2. During reconstruction of the orbital floor in patients of group 1, successful results were achieved in 68.2% (15/22) of cases and 88.2% (15/17) in group 2, various complications were observed in the rest of cases.

Based on the analysis of surgical treatment results, there was developed a personalized approach to manufacturing of zygomatic bone and orbital floor implants using computer modeling and 3D printing technologies.

**Conclusion.:**

In contrast to the conventional methods, the use of digital technologies in the surgical treatment of post-traumatic deformities of ZOC allows avoiding the problematic issues of implant positioning and the development of complications during reconstruction, significantly reducing surgical injury and improving patient rehabilitation.

## Introduction

One of the main tasks of the surgeon performing operations in the middle face is to increase treatment efficacy and improve the quality of life in patients after injury. In injury of the zygomatico-orbital complex (ZOC), especially, that with pronounced dislocation of fragments, delayed or unplanned surgery or its absence can lead to aggravation of injuries to the orbit, eyeball, oculomotor muscles, orbital fat, and the optic nerve. As a result, patients develop persistent post-traumatic deformities of the zygomatic area and the orbit, functional and aesthetic disorders requiring long-term multi-stage treatment [1–3]. According to various data [2–4], fractures of the zygomatic bone account for 20 to 37.5% of all injuries to the bones of the facial skeleton and 35–40% of these are orbital floor fractures.

Orbital fractures disrupt the anatomy and physiology of the orbit, which lea

ds to ophthalmic and neurological disorders (deformity and change in the volume of the orbit, diplopia, restriction of eye movement, lost or impaired vision, hypoesthesia of the infra-orbital nerve) developing in the early post-traumatic period. Later, in the absence of treatment, hypo- and enophthalmos are likely to develop, diplopia, and cosmetic disorders progress [4–6]. These changes make it possible to consider ZOC injuries the most complicated in terms of reconstruction and restoration of impaired functions.

To date, the optimal algorithm for the management of patients with ZOC fractures and deformities has not been developed: various surgical approaches and techniques are used, a variety of materials and implants are employed to reconstruct the zygomatic bone and the orbital floor [5, 7—9]. The gold standard for the surgical treatment of injuries of the zygomatic bone and orbital floor is the use of autobone, though polymer implants, metal plates, and mesh are widely used as well [2, 3, 8–12].

All of the above pertains to conventional (classical) surgical treatment methods for patients with injuries of the ZOC, but they have certain advantages and disadvantages. The disadvantages include an increase in the duration of surgical procedure due to autobone sampling or intraoperative modeling of metal and polymer prostheses; autobone resorption; hyper- or hypocorrection of the zygomatic area; persistence of hypophthalmos or enophthalmos; orbital fat atrophy; the development of metallosis or titanium implant eruption; persistence of functional and cosmetic problems [3, 6, 12–14].

The first decade of the XXI century is distinguished by active introduction of digital technology in the work of maxillofacial surgeons. This is mainly attributable to the emergence of new computer modeling methods, increasing availability and lower cost of equipment for visualizing CT and MRI data, the development of additive manufacturing and 3D printing of individual implants to restore the anatomy of the bone structures of the facial skeleton [3, 15—18]. Thorough preoperative preparation of a patient, virtual planning of the intervention, and the manufacturing of patient-specific implants make it possible to perform reconstructive plastic surgery on the ZOC using minimally invasive technologies [17, 18].

**The aim of the study** was to determine the efficacy of using digital technologies in patients with post-traumatic deformities of the zygomatico-orbital complex by comparing the results with the conventional methods of surgical treatment.

## Materials and Methods

The paper outlines the experience in the surgical management of 231 patients with ZOC injuries who underwent surgery at the clinical facilities of the Department of Surgical Dentistry and Maxillofacial Surgery with the Course of Plastic Surgery at Privolzhsky Research Medical University (Nizhny Novgorod) in 2011–2019. Those included the University Clinic, maxillofacial surgery departments of N.A. Semashko Nizhny Novgorod Regional Clinical Hospital and Clinical Hospital No.3, Privolzhsky District Medical Center of Federal Medico-Biological Agency of Russia. The average age of the patients was 33.6 years (20 to 60 years). There were 80% (188/231) of males and 20% (43/231) of females among them. Written informed consents from the patients for the use of data and publishing the photographs are available in medical records.

Timing for surgical treatment varied: 1.7% (4/231) of patients underwent surgery on day 1 after the injury, 19.5% (45/231) — on days 7 to 14, 33.8% (78/231) — on days 15 to 30, 27.3% (63/231) — after 1–2 months, 12.5% (29/231) — after 4 months, 4.3% (10/231) of patients were operated on after 6 months. Of all the examined patients with post-traumatic deformities of ZOC, 44.2% (102/231) underwent treatment. Of them, 38.2% (39/102) had post-traumatic defects and deformities of the orbital floor. All patients had deformities of the zygomatic area and orbit, hypophthalmos and/or enophthalmos, diplopia of various types.

All patients underwent standard clinical examination and multispiral computed tomography (MSCT) of the skull in the axial, sagittal and frontal planes, 3D CT models being constructed before and after surgery. Ophthalmologists performed examination of patients before and after surgery: within 1, 3, and 6 months. Visometry, tonometry, ophthalmoscopy, and biomicroscopy were performed. The axial position of the eye in the orbit (eno- or exophthalmos) was identified using the Hertel exophthalmometer (Inami, Japan). Hypophthalmos was evaluated with respect to the horizontal line passing through the center of the pupil of the healthy eye. The characteristic aspects and size of binocular diplopia zone were determined using the Goldman spheroperimeter [3]. The above diagnostic data were absolutely necessary when planning the surgical treatment and evaluating the results of treating the orbit [1, 3, 6].

To eliminate post-traumatic deformity of the zygomatic area, classical surgical operations (osteotomy and osteosynthesis) were performed in 85.3% (87/102) of cases, while 14.7% (15/102) of patients underwent only contour plastic surgery of the zygomatic area with implants and reconstruction of the orbital floor. For post-traumatic defects and deformities of the orbit, orbital reconstruction was performed using Reperen polymer implants (Russia) in 38.4% (15/39) of cases, Konmet metal implants (Russia) in 30.8% (12/39) of cases, and Synthes implants (Switzerland) of standard sizes in 30.8% (12/39) of cases [12–14, 16].

To assess the role of X-ray planning of surgery for correcting post-traumatic deformities of ZOC, two groups of patients were formed. Group 1 (54.9% (56/102) of cases) included patients who underwent surgery without preoperative virtual planning. Group 2 (45.1% (46/102) of cases) included patients who underwent virtually planned surgical interventions. There were 22 and 17 patients with orbital deformities in groups 1 and 2, respectively.

Virtual planning was carried out using multi-planar reformation of CT images and performing three-dimensional reconstructions. We studied the specific features of post-traumatic deformities of ZOC. We determined the degree of bone structure dislocation and the size of the bone defect in the orbital floor, detected concomitant changes in the orbit, measured the linear dimensions of injured and intact orbital walls, assessed the degree of eyeball dislocation and determined the optimal position of the future implant.

In the postoperative period, all patients were administered standard medical treatment. Sutures were removed in 5–7 days after surgery. The follow-up examination of patients was carried out in 1, 3, 6 months and 1, 3, 5 years after surgery.

**Statistical processing** of the digital data was carried out using Microsoft Office Excel 2010 software, Statistica 10.0 software package (StatSoft, Inc.) in accordance with currently accepted methods of statistical analysis. Nonparametric Mann–Whitney U-test for unrelated groups was used when the values of the analyzed data did not correspond to the normal distribution law.

## Results and Discussion

Based on the analysis of surgical treatment results in 231 patients with injuries of the ZOC, we identified a group of patients (n=102) requiring surgical treatment for post-traumatic deformity of ZOC, of which 39 individuals required reconstruction of the orbital floor. Assessment of surgical treatment results in patients with post-traumatic deformity was carried out on the basis of clinical and radiological data and analysis of postoperative complications.

**Figure 1:**
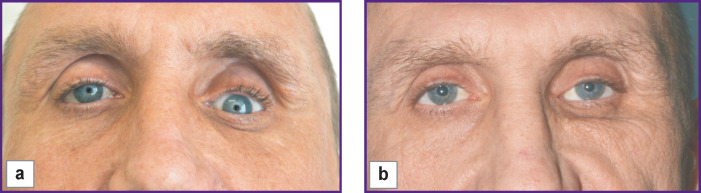
Patient K. Post-traumatic deformity of the left zygomaticoorbital complex, pronounced left hypo- and enophthalmos: (a) before surgery; (b) after surgical treatment

**Figure 2:**
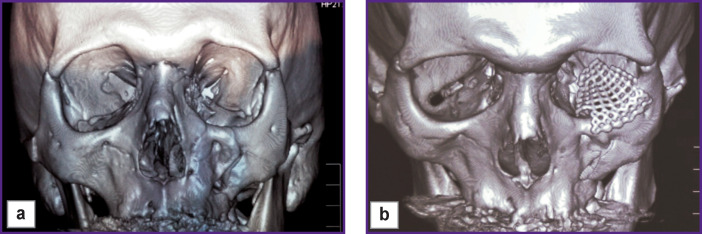
Patient K. 3D CT reconstruction of the skull: (a) before surgery; (b) after surgery (a titanium mesh implant installed on the left orbital floor and the infraorbital rim, partly on the body of the zygomatic bone)

**Figure 3:**
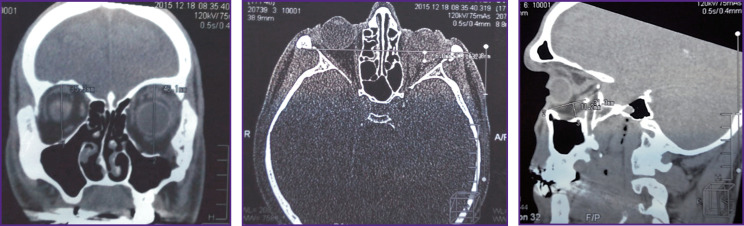
Patient K. MSCT of the skull before surgery (frontal, axial, and sagittal sections)

**Figure 4:**
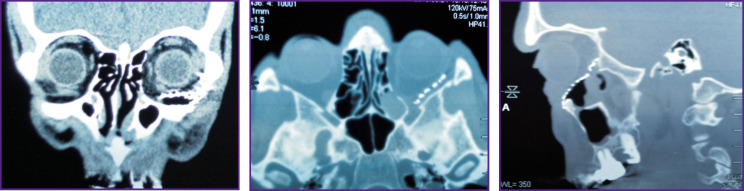
Patient K. MSCT of the skull after surgery (a titanium implant is visualized on the frontal, axial, and sagittal sections)

The optimal restoration of ZOC anatomy was observed in 75% (42/56) of patients in group 1 (without virtual planning of surgery) and 93.5% (43/46) of patients in group 2 (Figures 1–4). After reconstruction of the orbital floor, successful results were achieved in 68.2% (15/22) of patients in group 1 and 88.2% (15/17) in group 2, various complications were observed in other cases.

In the long-term postoperative period, 3 of 22 patients in group 1 had hypophthalmos and enophthalmos (3–4 mm) and 1 individual had hypophthalmos (5 mm). When analyzing MSCT of the orbits in these patients, prolapse of the distal edge of the implant towards the maxillary sinus was detected, therefore reoperation was required.

In the long-term period, hypophthalmos and enophthalmos (3–4 mm) were observed in 1 of 17 patients in group 2, which also led to recurrent operation. Enophthalmos limited to 1–2 mm caused no discomfort to patients of both groups, so there was no surgical treatment.

Three months after the intervention, diplopia persisted in 4 of 22 patients in group 1 and 2 of 17 patients in group 2. The restoration of their binocular vision took as long as 6 months, which was associated with the specificity of the injury and the timing of surgical treatment.

Patients with post-traumatic orbital deformity had various degrees of hypophthalmos and enophthalmos severity (see the Table).

**Table:** Distribution of patients according to the degree of orbital displacement before and after surgery

Degree of clinical manifestation	Hypophthalmos				Enophthalmos			
	Group 1 (n=22)		Group 2 (n=17)		Group 1 (n=22)		Group 2 (n=17)	
	before	after	before	after	before	after	before	after
0 (0 mm)	4	14	3	15	4	12	3	14
1^st^ (1–2 mm)	9	4	4	1	9	7	4	2
2^nd^ (3–4 mm)	7	3	6	1	7	3	7	1
3^rd^ (≥5 mm)	2	1	4	0	2	0	3	0

When analyzing long-term treatment results, we found no vision loss or inflammatory changes around the implants. Hyperophthalmos was observed in 1 of 22 patients in group 1 and 1 of 17 patients in group 2, and these patients had severe post-traumatic hypophthalmos and enophthalmos. During the surgery, they underwent moderate vertical overcorrection with metal implants in order to stabilize the position of the eyeball in the long term. In these patients, vertical eye position came back to normal 3 months after rehabilitation.

Implant displacement was observed in 2 patients from group 1 within 6 months after surgery. This complication was caused by the presence of a wide defect in the orbital floor, cicatricial changes in the orbital tissue, and displacement of the polymer implant due to scarring of the tissues into the maxillary sinus. Patients required repeated surgical intervention following the development of progressive diplopia. Analysis of reasons for implant displacement showed errors in planning the surgery and making decisions on the types and sizes of the implant that failed to provide sufficient bone defect coverage and support rigidity in the distal zone. There was no displacement of implants towards the lower eyelid as all the polymer and metal structures used were reliably fixed to the underlying bone with micro-screws.

In this study, we have improved the methodology for the analysis of diagnostic images during radiologic examination of patients with injuries and developed a personalized approach to manufacture of implants. In practice, the technology has now been tested in 2 patients with severe post-traumatic deformity of ZOC due to displacement of fragments and presence of wide bone defects in the orbital floor. Surgery was performed using two individual implants of the zygomatic bone and the orbital floor manufactured using computer modeling and 3D printing.

**Figure 5:**
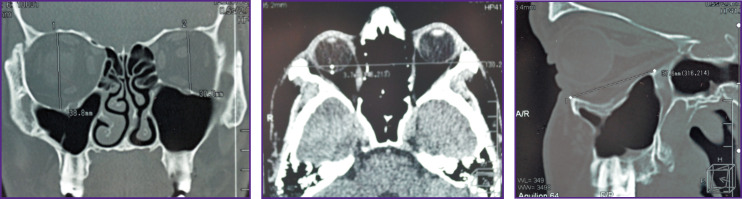
Patient N. MSCT of the skull before surgery (frontal, axial, and sagittal sections)

The use of standard implants in these patients was associated with a high risk of precision mismatch in the volume parameters of bone defects and the implants; pronounced displacement of fragments and deformity due to injury age; possible loss of correction in the postoperative period; persistence of diplopia and enophthalmos (Figure 5).

When planning surgical reconstruction of the ZOC, modern digital technologies were used to calculate the sizes of defects and deformities, their volumetric parameters and determine the shape of future implants.

*The first stage* involved creating a 3D computer model of the facial skeleton in accordance with MSCT data of the patient’s head, using 3D Slicer software.

*The second stage* consisted of hybrid parametric modeling with the use of the Autodesk Meshmixer software, deformity correction, replacement of bone defects using a “mirror copy” of the intact contralateral side of the skull.

**Figure 6:**
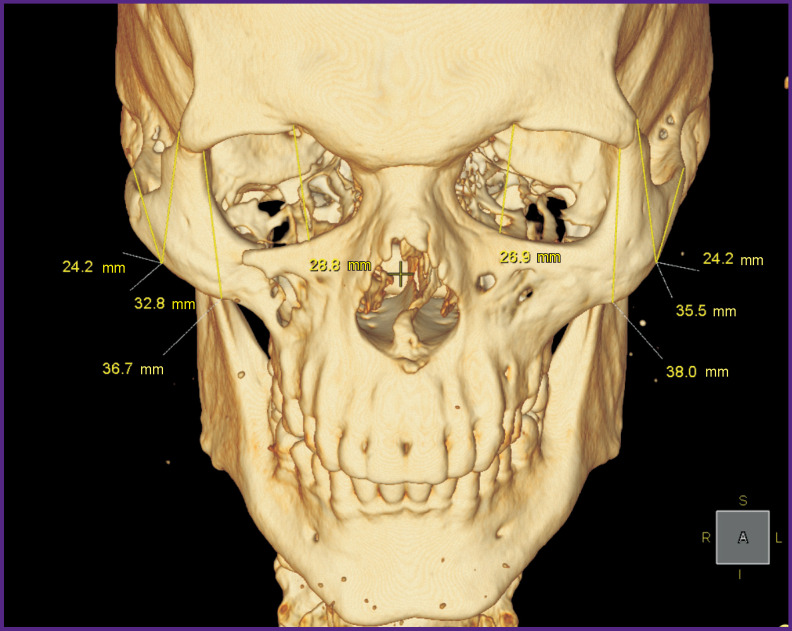
Patient N. 3D CT reconstruction of the skull. Stage of computer planning of surgery and implant modeling

During *the third stage*, three-dimensional models of individual implants were created and their topological optimization was performed for their subsequent manufacturing (Figure 6).

**Figure 7:**
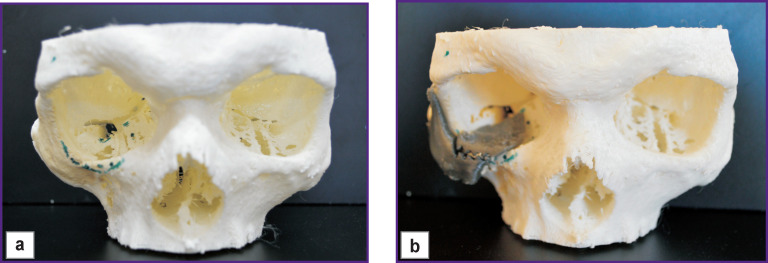
Patient N. Stage of surgery simulation using the model of the patient’s skull and two prototypes of individual implants: (a) before the installation of individual implant prototypes; (b) after installing the prototypes

The implant for reconstruction of the zygomatic bone was modeled taking into account the specific characteristics of the post-traumatic zygomatic deformity, decompression of the infra-orbital nerve and obligatory application of supports for connection with the orbital implant. Its thickness varied in the range between 0.1 and 0.8 mm, depending on the reconstruction region. The implant for reconstructing the orbital floor covered the bone defect completely, rested distally on the orbital eminence, and had the required thickness to eliminate enophthalmos. Next, 3D model of the skull and two prototypes of individual implants were printed. After that, they were used to simulate the operation using standard tools (Figure 7).

**Figure 8:**
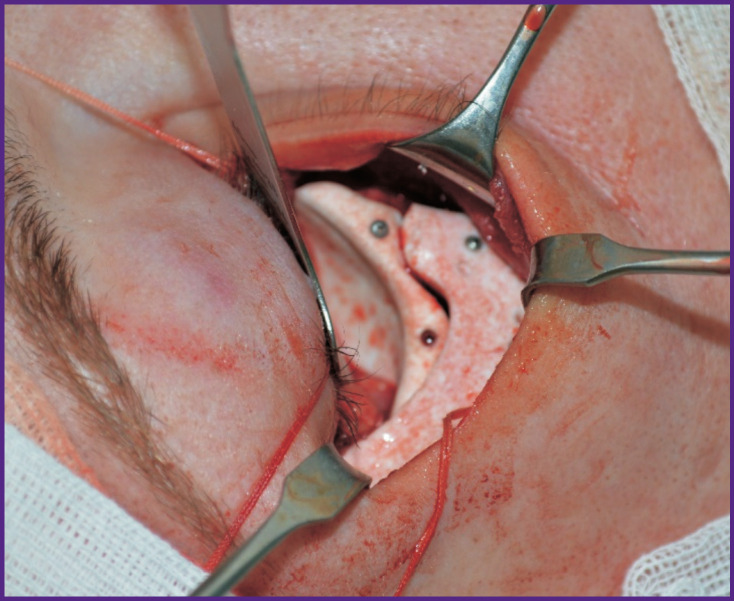
Patient N. Fragment of the surgical operation. Two polymer implants were installed: on the right orbital floor and the zygomatic bone

Further, polytetrafluoroethylene implants were manufactured based on the prepared prototypes of individual implants by Ecoflon (Saint Petersburg, Russia). During surgery, these implants were fixed to the underlying bone structures with titanium micro-screws (Figure 8).

**Figure 9:**
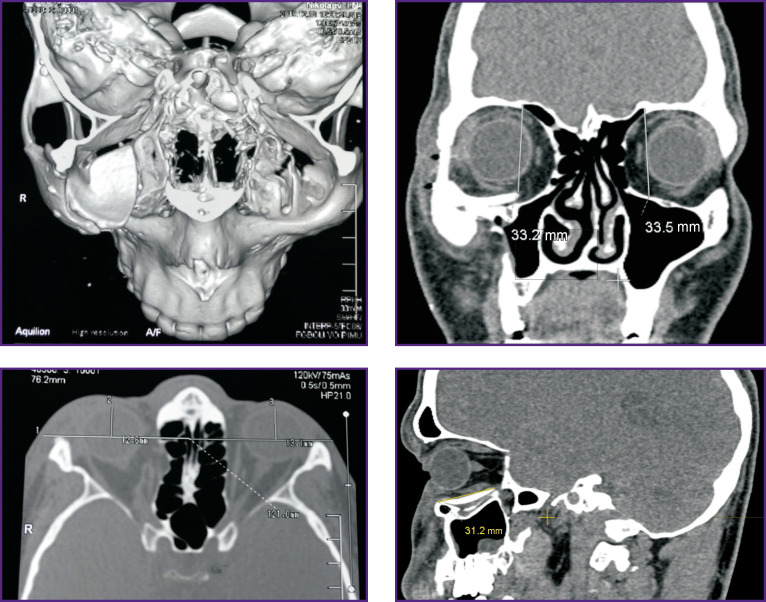
Patient N. MSCT of the skull after surgery. The polymer implant on the right orbital floor is visualized

**Figure 10:**
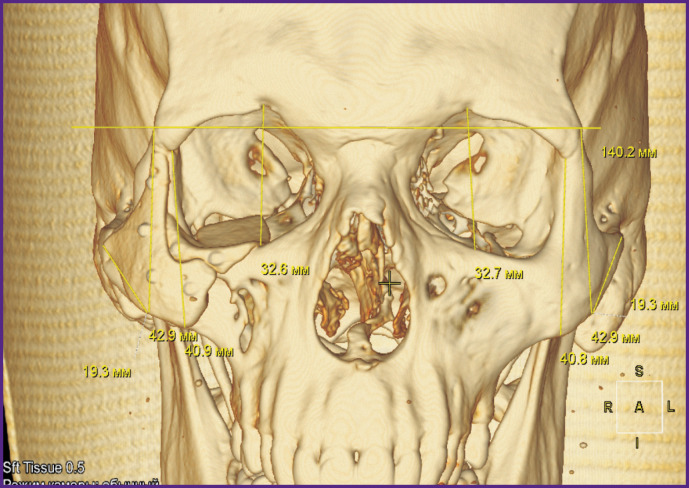
Patient N. Computer 3D model of the skull after surgery

The postoperative period was uneventful. All implants integrated well with the tissue, there was no suppuration or implant failure (Figures 9, 10).

**Figure 11:**
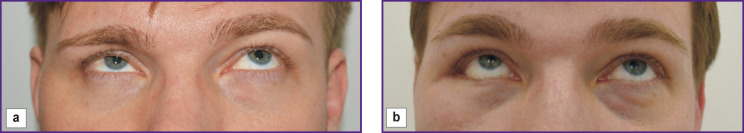
Patient N. Post-traumatic deformity of the left zygomatico-orbital complex: (a) before surgery; (b) 1 month after surgery

The use of digital technologies in surgical treatment of patients has made it possible to optimally correct post-traumatic deformities of ZOC, increase the accuracy of orbital floor reconstruction, reduce the duration and extent of surgical intervention, and obtain predictable results (Figure 11).

## Conclusion

In contrast to the conventional methods, the use of digital technologies in the surgical treatment of post-traumatic deformities of the zygomatico-orbital complex allows avoiding the problematic issues of implant positioning and the development of complications during reconstruction, significantly reducing surgical injury and improving rehabilitation of patients.
